# Fifteen Years of Airborne Particulates *in Vitro* Toxicology in Milano: Lessons and Perspectives Learned

**DOI:** 10.3390/ijms21072489

**Published:** 2020-04-03

**Authors:** Eleonora Marta Longhin, Paride Mantecca, Maurizio Gualtieri

**Affiliations:** 1 Health Effects Laboratory, Department for Environmental Chemistry, NILU–Norwegian Institute for Air Research, Instituttveien 18, 2007 Kjeller, Norway; eml@nilu.no; 2 Department of Earth and Environmental Sciences, Research Center POLARIS, University of Milano-Bicocca, 20126 Milan, Italy; paride.mantecca@unimib.it; 3 ENEA SSPT-MET-INAT Bologna Research Centre, Via Martiri di Monte Sole, 4, 40129 Bologna, Italy

**Keywords:** PM_10_, PM_2.5_, ultrafine particulate matter (UFP), inflammation, oxidative species, DNA damage, epigenetic modification

## Abstract

Air pollution is one of the world’s leading environmental causes of death. The epidemiological relationship between outdoor air pollution and the onset of health diseases associated with death is now well established. Relevant toxicological proofs are now dissecting the molecular processes that cause inflammation, reactive species generation, and DNA damage. In addition, new data are pointing out the role of airborne particulates in the modulation of genes and microRNAs potentially involved in the onset of human diseases. In the present review we collect the relevant findings on airborne particulates of one of the biggest hot spots of air pollution in Europe (i.e., the Po Valley), in the largest urban area of this region, Milan. The different aerodynamic fractions are discussed separately with a specific focus on fine and ultrafine particles that are now the main focus of several studies. Results are compared with more recent international findings. Possible future perspectives of research are proposed to create a new discussion among scientists working on the toxicological effects of airborne particles.

## 1. Air Pollution in Urban European Cities: The Case Study of Milan

Milan is the biggest city in the Po Valley area with 1.3 million inhabitants, the second largest in Italy after Rome, and one of the most populous metropolitan cities in Europe with more than 7.5 million inhabitants (considering the Milan Metropolitan area). Air pollution in the Po Valley region is a matter of concern. The latest air quality report from the European Environment Agency (EEA) [1] shows that, in 2016, the majority of monitoring stations of the Po Valley registered a mean annual concentration of particulate matter, with mean aerodynamic diameter lower than 10 µm (PM_10_), higher than the annual threshold of 40 µg/m^3^. Similar results were also found from the monitoring stations located in Milan Metropolitan Area, where particulate matter (PM) still represents one of the most critical environmental issues for its impact on human health [2]. Since the 1990s, local, regional, and national authorities have made efforts to reduce airborne particles concentration, and significant improvements were achieved. Nonetheless, in the last decade, the rate of reduction of air pollution over the Po Valley region slowed [3,4], mainly due to the geomorphological condition of this area. In fact, the rate of reduction remained significant at urban stations and was mainly associated with the renewal of circulating vehicle fleets [3]. The latest data available for the city of Milan (data from the EEA website, https://www.eea.europa.eu/data-and-maps/data/aqereporting-8#tab-figures-produced and http://discomap.eea.europa.eu/map/fme/AirQualityExport.htm and https://www.eea.europa.eu/data-and-maps/indicators/exceedance-of-air-quality-limit-3/assessment-5) confirm that annual averages of fine particulate matter, i.e. particles with aerodynamic diameter lower than 2.5 µm (PM_2.5_), measured at an urban traffic (Via Senato) and at an urban background (Via Pascal, Città Studi) station, are not significantly different. These data also show that the annual limit of 25 µg/m^3^ set by the European Union (EU) Directive 2008/50/EC is not always respected. Therefore, due to its peculiar geomorphological conditions, and as the biggest city in the north of Italy, the city of Milan represents a significant case study at the European level for comprehension of the potential impacts of air pollution in an exposed population [2]. In this context, in 2008, a research center on Particulate Matter and Health Risk (POLARIS) was instituted at the Department of Environmental Sciences (now Dept. of Earth and Environmental Sciences) of the University of Milano-Bicocca. POLARIS is a multidisciplinary scientific body for chemical, physical, biological, toxicological, clinical, and epidemiological research on air pollution in Milan, and more in general, in the Lombardy region and Italy. Over the last twelve years of activity, POLARIS researchers uncovered and published significant and interesting processes related to air pollution, including particulate matter (PM) chemical and microbiological characterization, *in vitro* and *in vivo* toxicological effects, as well as clinical and epidemiological studies [5,6,7,8]. 

Nowadays the presence of micro and nano-plastic debris in the environment is attracting increasing scientific interest. A recent study [9] reported the presence of airborne microplastics in European and Chinese cities and regions, with fragments and fibers derived from the wearing out of plastic goods (i.e., synthetic textiles, waste treatments, industrial emissions, etc.). In addition to these new emerging plastic particles, the wearing out of tires is nowadays (re)considered as a significant emission source of plastic particles [10,11] with an estimated contribution of 3%–7% to PM_2.5_.

This review summarizes the most relevant results obtained through *in vitro* studies on the toxicological impact of airborne PM, also considering the international literature, and proposes the focal research points that, in the coming years, researchers, who deal with the mechanisms and modes of action related to airborne pollution and health risks, should take in consideration. 

## 2. Coarse PM and PM_10_

PM_10_ was the first fractional dimension subject to EU environmental regulation, with limits for atmospheric concentrations since 1999 (First Daughter Directive to the Air Quality Framework Directive). As a consequence, it was also the fraction that first received attention from the community of environmental toxicologists. However, in the last years, the interest on PM_10_ toxicological potential has received less and less attention if compared to fine PM (i.e., particulates with an aerodynamic diameter lower than 2.5 µm (PM_2.5_)), and ultrafine PM (i.e., particulates with an aerodynamic diameter lower than 100 nm, known as PM_0.1_ or ultrafine particulate matter (UFP, i.e. particles with aerodynamic diameter lower that 100 nm)). 

The reasons for this reduced research interest stand in the increased number of studies focusing on the effects of selected emission sources (vehicular emissions, biomass burning, ship emissions, etc.) and by the increasing evidence from epidemiological studies showing a strong correlation between fine PM exposure and adverse health effects [12,13]. Nonetheless, a clear relationship between coarse PM and biological effects, in exposed models *in vitro* and *in vivo*, has been largely reported [14,15]. Indeed, the capability of coarse PM to induce inflammation in cells has been associated to the higher content of crustal elements and fragments from bacteria and other biological components. The question, largely discussed, is at what extent are PM_10_ and coarse PM (i.e., PM fraction with mean aerodynamic diameter lower than 10 µm and higher than 2.5 µm (PM_10-2.5_)) relevant to explain adverse human health effects. Although out of the scope of this review, it is worth mentioning that coarse airborne particles deposited in the tracheobronchial respiratory tract may end into the gastrointestinal system [16,17]. Cilia of bronchial cells are the engine of the mucociliary escalator [18] that transports the mucus, and the particles trapped in it, from the lungs up to the larynx where it is swallowed. The particles thus enter the gastrointestinal (GI) system where they may interact with GI tissues and fluids [19,20,21]. In the alveolar region particles may be actively removed by specialized cells (macrophages) and other mechanisms (reviewed in [22]). Considering the deposition curves of particulates in the lung, larger PM_10_ particles should mainly deposit in the upper respiratory tract affecting the bronchial and thoracic regions [23]. The effects of these particles on nasal and bronchial epithelial cells have been therefore considered relevant for the assessment of toxicological effects of airborne PM_10_. The reported effects, which include inflammation, oxidative stress, DNA damage, and cell death, have been related to the activation of different biological pathways [24,25,26]. Given the significant difference registered in Milan PM chemical composition during cold and warm seasons [8,27], an evaluation of the biological effects of PM_10_ was performed comparing the potency of samples obtained during winter and summer seasons. The experience collected over a decade showed two main different impacts of these particulates: summer PM_10_ was able to induce an increase in inflammatory mediators in the exposed *in vitro* systems [14], also activating the nucleotide-binding domain, leucine-rich-containing family, pyrin domain-containing-3 OR Nod-like receptor protein 3 (NLRP3) inflammasome and, consequently inducing the release of interleukin (IL)-1β [28]. On the contrary winter PM_10_ was a more potent inducer of DNA damage and reactive oxygen species (ROS) formation [29]. These differences were related to the chemical composition of the particles, with summer PM enriched in crustal elements and pro-inflammatory biogenic compounds [14,25], and winter PM characterized by a higher content in organic compounds (such as polycyclic aromatic hydrocarbons, PAHs) [14]. These results are in agreement with the international literature [26,30,31,32,33,34,35]. Indeed, the toxicological characterization of PM_10_ and coarse PM has received significant interest during the last decade of 20^th^ century and the beginning of 21^st^ century. The capability of PM_10_ to induce inflammation has been identified by several authors [36,37,38,39] and related to different PM components, such as endotoxins [30,31,32], metals and crustal elements [26,30,34], and possibly PAHs [33,35]. This evidence comprises the first lesson learned: an often neglected component of airborne PM i.e. biogenic-derived debris, which is seldom characterized, and natural occurring species (crustal elements) may trigger significant lung inflammation. This may also support the epidemiological evidence reporting increased mortality and hospitalization during summer peaks of air pollution for cardiopulmonary diseases [40,41,42]. However, the toxicological difference in PM samples from different seasons is not supported by all authors. For example, Dumax-Vortex et al. [43] showed similar elemental composition and biological effects for particles sampled in Manchester (UK) during summer and winter season, indicating that the seasonality might not be a relevant parameter in every region.

**Figure 1:**
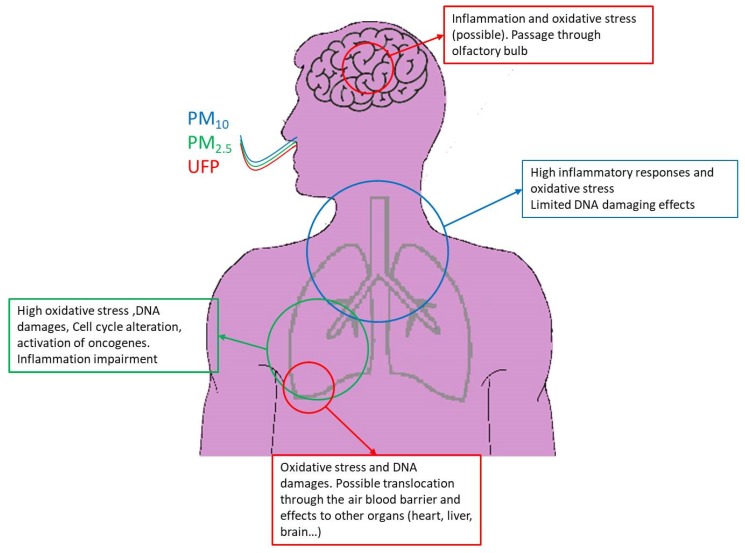
Summary of adverse effects determined in *in vitro* systems exposed to particulate matter with mean aerodynamic diameter lower than 10 µm (PM_10_), fine particulate matter, lower than 2.5 µm (PM_2.5_), and ultrafine particle matter (UFP). PM_10_ coarse particulates deposit in the upper respiratory tract eliciting mainly inflammatory responses and formation of oxidative species. Particles rich in organic components may also trigger DNA damage and related responses. Fine PM deposits deeper in the respiratory tract and induces an increase in intracellular oxidative species. Consequent oxidative DNA damage and DNA damage adducts are related to xenobiotic responsive element (XRE) and antioxidant responsive element (ARE) genes expression. UFP deposit in the nose from where they may translocate to the brain through the olfactory bulb, inducing oxidative stress and inflammation in neural and glial cells. UFP may reach the alveoli from where they may translocate into the blood and target secondary organs such as the heart, brain, liver, etc.

The capability of PM_10_ to induce damages to the DNA, oxidative species, and activation of the xenobiotic responsive element (XRE) is also a well-learned lesson (already reviewed in [44] and related to the content of organic compounds, such as PAHs and dioxins). An interesting study [45] reported the capability of PM_10_ to impair the antioxidant defense system in lung cells, therefore increasing the possibility of oxidative damages and eventually cell death. The relevance of PM_10_ to induce reactive species (both oxygen and nitrogen ones) is reviewed in detail by Donaldson and colleagues [24]. Other papers [46,47,48] reported clear association between PM_10_ oxidative properties and DNA damage. More recently a study by Morales-Bárcenas and co-workers [49] reported an alteration induced by PM_10_ on protease activity and cell invasion. The authors also concluded that this alteration, combined with inflammatory insults also promoted by airborne PM, is likely to increase the possibility of chronic lung diseases, including cancer. Accordingly, Quezada-Maldonado and colleagues reported [50] a PM_10_-associated deregulation of micro-RNAs (miRNAs) relevant for cellular pathways usually modified in cancer cells. In this context, future research should focus on the consequence of long-term exposure to PM from different seasons and with clearly different toxicological potency, for example, summer (inflammogenic) and winter PM_10_ (genotoxic) (Figure 1). 

## 3. Fine PM (PM_2.5_) 

Although an EU daily limit for PM_2.5_ exposure is not in operation (the EU directive 2008/50/EC refers to an annual mean of 20 µg/m^3^ in force from 1 January 2020), the World Health Organization (WHO) recommends a 24 h threshold of 25 µg/m^3^ (with an annual average of 10 µg/m^3^) for protection of human health. Despite this lack of a daily exposure limit, the EEA (https://www.eea.europa.eu/data-and-maps/indicators/exceedance-of-air-quality-limit-3/assessment-5) also reports that 6%–8% of the urban population are exposed to concentrations in excess of the EU target value of 25 µg/m^3^, while 74%–81% are exposed to concentrations above the WHO recommended annual concentration. The significance of these exceedances is related to the latest epidemiological reports that identify fine PM as the fifth leading risk factor for death in the world, accounting for 4.2 million deaths and more than 103 million disability-adjusted life years lost [51,52]. 

It is therefore straightforward to understand the strong effort that has been devoted by many research groups in Europe and all over the world in identifying the key biological processes activated by PM_2.5_. The research conducted on Milan fine PM unraveled significant biological outcomes, which were only in part previously reported. Significantly, we show the capability of airborne fine PM to interact and impair one of the key cell processes that is cell division [53,54], by altering the spindle assembly during prophase and metaphase. Although working on PM_10_, a recent study by Santibáñez-Andrade and co-workers [55] demonstrated that PM-induced cell division impairment is related to the disruption of the spindle assembly checkpoint. A correct cell division is essential to ensure that the replicated chromosomes are evenly separated in the daughter cells. In accordance, we reported that fine PM exposure increased the number of cells with micronuclei [53], providing additional evidence that relates PM exposure, genomic instability and, possibly, lung cancer onset (as reviewed in [56]). A typical feature of lung tumor cells is their ability to invade surrounding tissues and produce metastasis. Invasion potency has been related to cytoskeleton remodeling [57]. We extensively report that PM_2.5_ is able to determine significant modifications in the cell cytoskeleton, such as loss of stress fibers, modified actin structure, and increased membrane ruffling [54,58]. These data are supported by a recent publication by Chirino and colleagues [59]. These alterations were related to a significant relocation of cadherin-1 and increased serpin-2 expression and heat shock protein 27 (HSP27) phosphorylation [58,60]. Furthermore, we and others showed that cytoskeleton alteration is also associated to pro-inflammatory signaling impairment in exposed lung cells [61,62], which may increase the susceptibility of lung tissue to infection and other diseases. Taken together, these results evidence how exposure to fine PM potentially contributes to all phases of the carcinogenic process. In addition, fine winter PM is able to activate a series of different pathways related to aryl hydrocarbon receptor (AhR) activation, nucleotide-binding oligomerization domain (NOD)-like receptor activation, and reactive oxygen species formation leading, among others, to XRE genes (*Cyp1A1*, *Cyp1B1*, *AhRR*) and antioxidant responsive element (ARE) genes (*NQO1, HMOX-1, SOD, GCL, ALDH3A1*) transcription, which eventually determines the increase of related protein levels [58,63]. The effects, reported by our studies, are associated also with increased DNA damage, mainly oxidative, as a result of the increased formation of ROS. Indeed, the increase in measured ROS after PM exposure correlated with γH2AX and 8-oxodG levels [53]. Increments of RNA levels of genes related to XRE and ARE are not new, and other authors reported this association with fine PM. The differential modulation of genes in cells exposed to PM_2.5_ samples, collected during different months and at different locations, is reported by Lauer and colleagues [64] and shows an increased expression of genes related to AhR activation (*Cyp1A1*) and oxidative stress (*HMOX-1, NQO-1, ALDH3A1, AKR1C1*). Similarly, another study by Zhou and collaborators [62] shows increased expression of ARE related genes. The increased expression of *Cyp1A1*, *Cyp1B1*, and *AhRR* genes after exposure to fine PM, also in relation to increased γH2AX levels, is reported by Borgie and co-workers [65] and a recent study by Mehta and colleagues [66] shows that PM is also able to impair the DNA repair mechanisms, therefore increasing PM mutagenic effects [67,68]. PM-derived activation of AhR was also shown to activate dendritic cells [69], which are essential in priming naïve T cells, therefore providing additional evidence relating airborne PM and immune responses. In this respect, it is pivotal to remember the interplay among the different response pathways within cells, as demonstrated by authors working on specific fine PM compounds, such as PAHs [33,70] (Figure 1). 

The importance of epigenetics on the onset of several human diseases is largely documented [71,72,73,74] and, in this context, fine PM shows to significantly modulate micro RNAs (miRNAs). We reported the modulation of several miRNAs, among which miR-1246 and miR-146a were shown to be upregulated [58]. miR-146a is reported to be relevant for several non-transmissible and autoimmune human diseases [75,76]. Recently, the importance of long non-coding RNAs (lncRNAs) has been reported to explain the effects of PM_2.5_, thus demonstrating the complexity of interactions among cellular pathways activated by particles. A recent study by Li et al. [77] related lncRNAuc001.dgp.1, targeting miR-3607-5p, to the inflammatory properties of fine PM. Another study published in 2017 by Deng et al. [78] correlated the ability of PM_2.5_ to induce autophagy and cancer cell migration and invasion with increased expression of lncRNA loc146880. In 2017 Xu et al. [79] showed that lncRNA LINC00341 is involved in PM-induced cell cycle arrest in G2/M phase. Interestingly, a more recent study by Xu et al. [80] reported the modulation of several exosome-related miRNAs, after exposure of bronchial cells to fine PM. In this respect, a few authors have already pointed out the importance of PM in modulating the release of exosomes/macrovesicles in exposed *in vitro* models [81,82], also in view of the potential role of these extracellular structures in mediating human diseases [83,84,85] (Figure 1). 

Finally, in addition to the direct effects of fine PM on lung epithelial cells, authors are reporting interesting findings on the interaction of particulates with lung fluids. A study published in 2016 by Zhou et al. [86] reported the interaction of fine PM with lung fluids and demonstrated significant adverse effects on a cell membrane model while a recent paper by Dean, Elom, and Entwistle [87] proposed that the interaction of particulate with lung fluid may increase the exposure to heavy metals in urban areas, suggesting another possible mechanism for PM-related health effects. 

## 4. Ultrafine PM 

Ultrafine PM particles have received significant interest from researchers in recent years. This is mainly due to the understanding that PM mass concentration and PM particle number concentration are driven by different PM fractions, with the coarse and fine fraction leading the first and ultrafine particles leading the latter. Furthermore, the discussion of the importance of UFP on the explanation of health outcomes related to air pollution is gaining importance among researchers [88].

Epidemiological analyses show a relationship between UFP number concentration and both myocardial infarction [89] and cardiopulmonary health [90], although other papers report no or limited associations of UFP number concentration with health diseases [91,92] or mortality [93]. These differences may be related to the time lag between exposure to UFP and health status assessment, or to differences in the chemical properties/sources of measured UFP [94,95]. It is therefore straightforward to understand the request made by several authors to implement real-world exposure protocols for toxicological models relevant to PM and UFP [96,97]. Real-world direct exposure and environmentally-relevant doses will allow understanding of the toxicological effects of, possibly, unbiased PM and UFP samples, although representative of a specific area. However, given the significant artefacts produced by the particulate sampling and the extraction procedures, normally used for toxicological tests, only the modification of the paradigm so far applied, that is, sampling–extraction–exposure, into a new one based on direct exposure, may help to understand in more detail why particulates from different places of the world are driving similar (or not) health responses in the exposed population. According to this new paradigm, we reported recently [98] the effects of fine PM and UFP on bronchial *in vitro* models exposed at the air–liquid interface (ALI) directly under environmental conditions. Our results showed, for the first time, the feasibility of using *in vitro* models for direct exposure to airborne aerosols, and the importance of UFP number and median diameter in triggering oxidative responses in exposed cells. Significant correlations were also found between black carbon and PAHs/organic matter fractional concentration and XRE related genes. These results agree with those reported in another study [99], which evaluated the capability of different PM_10_ size fractions to induce DNA damage in a cell-free system, showing that UFP was a potent inducer of damage. Moreover, the direct exposure allowed us to identify the relative importance of primary anthropogenic and secondary PM, and related sources of emissions, on the effects determined in exposed cells and to find interesting correlation with human biomarkers of exposure [98,100]. Significantly, our approach was performed without complex procedures aimed at replicating airborne PM [101,102]. Despite the intrinsic relevance of direct exposure approaches, the number of papers reporting them is still limited, particularly in relation to the significant number of online monitors required to characterize particles during exposure. Classical approaches, based on preliminary particles sampling, are therefore still largely applied, with subsequent ALI [103,104] or submersed exposure [60,105,106]. Since primary UFP sources are mainly related to combustion processes, we extensively analyze the toxicological effects of two main sources of fine and ultrafine PM, that are diesel and biomass combustion processes. Of relevance, we show [107] that, after only one hour of exposure, UFP induces complex transcriptomic modification relatable to possible cancer onset, as also reported by Longhin and collaborators [60]. These results are not new since the carcinogenic potency of diesel UFP is largely documented [108,109,110,111,112] but shed new light on the possible mechanism(s) of effects. In particular, we evidence that the epithelial–mesenchymal transition (EMT) may be regulated by *STAT3* and related genes (*HES1, HMOX1, IL6, IL24* and *VEGF*); the oncogene *KRAS* may be a key node in determining UFP effects by upregulating the expression of *EREG;* and finally, the hypoxia inducible-factor-1a (*HIF1a*) may play a central role in UFP-related endothelial dysfunction. Building up on these results, we also show the ability of UFP to induce endothelial modification after exposure of lung epithelial cells to UFP [113] suggesting the relevance of cell signaling on endothelium modification and the possible onset of relevant effects at the cardiovascular level. Again, the cardiovascular effects of combustion particles are well documented [114,115], but the importance of second messengers in determining the onset of these health issues is poorly understood. 

## 5. Future Perspectives

Given the increasing epidemiological and toxicological reports relating exposure to fine and ultrafine pollution with impairment of cognitive function [116,117,118,119], one of the primary areas for future toxicological studies should be the understanding of if and how UFP, after interaction with nasal and alveolar epithelial cells, may determine the activation of adverse cellular pathways in neuronal and glial cells [120]. So far the hypotheses are mainly related to the oxidative stress/inflammation paradigm, that will then determine significant damages in cells leading to cell modification and/or to cell death [121,122]. However it is not at all understood which component(s) of PM leads this process, if the carbonaceous core carbon of ultrafine PM is essential, if a specific class of compounds may be more likely responsible for adverse effects, or which processes are activated after exposure and, last but not least, if particle translocation occurs from primary (respiratory tract) to secondary target organs, such as the central nervous system (CNS), and the relevance of this process in triggering adverse effects. Therefore, we suggest that:

- the association of ALI exposure of lung cells to fine and ultrafine PM with neural cells, possibly in co-culture systems, is a primary goal in future toxicological studies for understanding the mode of action relating particulates with neurological effects.

The importance of understanding the biological effects of air pollution under realistic exposure conditions, which represent the real particle concentration exposure experienced by a population, has been largely evoked in recent papers [96]. Nonetheless the number of papers published during the last years is still scanty [98]. The reasons for this may be related to the complexity in setting up all the systems required for direct exposure (PM cut-off, particle diameters number distribution, ALI module, etc.) and for relevant chemical analysis (on-line vs. off-line procedures in relation to the time of exposure). Nonetheless ALI environmental exposure of lung epithelial models, possibly also in co-culture with other cell types, may allow the evaluation of selected PM fraction effects during specific hours of the day (i.e., commuting vs. working hours) and in different microenvironments. This new approach may also be used for repeated exposure experiments, taking advantage of the stability of ALI *in vitro* models during consecutive days. In this respect, we hope for:

- a significant increase of studies that will assess, using environmental exposure conditions, the association between PM and toxicological outcomes at doses and under sources directly representative of human exposure.

One of the most discussed questions among toxicologists is the identification of relevant correlation between different PM components and reported adverse effects. The correlations reported are always rather general: organic compounds, mostly PAHs, are associated with AhR activation and consequent Cyp enzymes’ expression with possible DNA damage [53,123]; PM dimension and surface properties, inorganic components (metals and ions), and PAHs are associated to ROS formation and consequent oxidative damage; elemental carbon/black carbon core is also associated with ROS formation. Nonetheless, PM is a highly complex matrix and different researchers already reported that compounds belonging to the same chemical class (i.e., PAHs also in their oxidized or nitric form) may counteract each other’s biological activities [33,124,125,126] competing for the same cellular substrate (like AhR), but triggering different biological responses (such as, but not limited to, XRE vs. inflammation). We therefore determine the following as important:

- the promotion and funding of a research effort (at European level at least) voted at the understanding of the molecules and, consequently, the sources of PM that are responsible of the observed toxicological effects, by means of a common platform for toxicological and chemical analysis, with the intent to obtain a common database on which to run data analysis. This information could, in turn, be useful for future air quality plans aimed at reducing the source of emission which impacts the health of exposed population more.

In recent years significant emphasis has been devoted to the effects of ultrafine carbon particles from primary combustion emission, namely vehicular emissions and, in part, biomass burning. Urban areas close to highways generally suffer from worse air quality, compared to inner city areas or low traffic zones [127,128]. This may be associated with higher impairment of health. Nonetheless, epidemiological studies are not able to properly consider these hot spots of exposure, mainly due to limited availability of personal exposure data. In the future we consider that

- the evaluation of the toxicological effects of micro areas, which are mostly impacted by vehicular emission, should also be considered for a more rationale planning of urban spaces and transports and for providing, in the end, equal access to clean and safe air to all of the population residing in a city.

In this respect, the fast moving transition of private mobility from internal combustion engine vehicles to full electric or hybrid vehicles requires a specific focus on the increasing incidence of non-exhaust vehicle emissions (such as brake wear, tire and road wear, etc.) on airborne PM [129,130,131]. Therefore a more focused evaluation of the toxicological impacts of fine and ultrafine particles deriving from these mechanical wear processes is desirable. 

Finally, new emerging pollutants/environments of exposure are calling for new toxicological data in order to provide evidence relevant for their correct risk assessment and management. In the future research specific effort should be devoted to defining the toxicological impact of particulates characteristic of indoor environments [132,133], new emission sources, such as e-cigarettes [134], and new emerging pollutants such as micro and nano-plastics [135,136,137].

Although the effects of indoor air pollution on health are of mounting interest, research should continue to investigate outdoor PM; especially considering that at regional and local levels, the rapid climate changes forecasted for the next decades are predicted to significantly affect air quality, including PM concentrations and chemistry, and the concentrations of other pollutants, like ozone and other gases, with expected strong impact on public health [138,139]. Again, this contributes to the importance of approaching the issue of air pollution-related health effects by newly designed toxicological studies. These studies should take into account the chemical transformation of fine and ultrafine PM under variable environmental parameters (i.e., temperature, UV light, humidity) and the association with other airborne pollutants, in order to investigate possible synergistic effects on respiratory and cardiovascular systems.
